# Frailty Diagnosed With the Clinical Frailty Scale Stratifies the Risk of Covert and Overt Hepatic Encephalopathy in Patients With Cirrhosis

**DOI:** 10.1002/jgh3.70369

**Published:** 2026-02-25

**Authors:** Shinji Unome, Takao Miwa, Sachiyo Hirata, Satomi Nakashima, Kayoko Nishimura, Mikita Oi, Masashi Aiba, Kenji Imai, Koji Takai, Masahito Shimizu

**Affiliations:** ^1^ Department of Gastroenterology/Internal Medicine, Graduate School of Medicine Gifu University Gifu Japan; ^2^ Department of Medicine University of California San Diego La Jolla California USA; ^3^ Center for Nutrition Support and Infection Control Gifu University Hospital Gifu Japan

**Keywords:** frail, liver disease, malnutrition, minimal hepatic encephalopathy, sarcopenia

## Abstract

**Aims:**

Frailty predisposes patients with cirrhosis to hepatic encephalopathy (HE). This study aimed to evaluate the effect of frailty on risk stratification for covert HE (CHE) and overt HE (OHE) in patients with cirrhosis.

**Methods:**

Hospitalized patients with cirrhosis and without history of OHE were retrospectively included. Frailty was assessed using the Clinical Frailty Scale (CFS). Factors associated with CHE and OHE development were evaluated using the logistic regression and Fine–Gray competing risk regression models, respectively.

**Results:**

Among 262 patients (median [interquartile range] age, 65 [55–74] years; 154 [58.8%] female), frailty and CHE were identified in 25 (9.5%) and 82 (31.3%) patients, respectively. The prevalence of CHE was higher in patients with frailty than in those without frailty (84.0% vs. 25.7%; *p* < 0.001). During a median follow‐up of 2.9 years, 40 patients (15.3%) developed OHE and 20 (7.6%) died. The incidence of OHE was higher in patients with frailty than in those without (incidence rates at 1, 3, and 5 years; 25%, 33%, and 36% vs. 5%, 11%, and 18%; *p* = 0.009). Multivariable analyses showed that CFS was an independent factor for CHE (odds ratio, 2.13; 95% confidence interval, 1.41–3.37; *p* < 0.001) and OHE development (subdistribution hazard ratio, 1.38; 95% confidence interval, 1.02–1.87; *p* = 0.037).

**Conclusion:**

Frailty assessed using the CFS is a robust factor to stratify the risk of CHE and OHE development in patients with cirrhosis. Patients with frailty should be screened and carefully monitored for HE.

**Abbreviations**
ALBI: albumin‐bilirubin
CFS: clinical frailty scale
CHE: covert hepatic encephalopathy
CI: confidence interval
HCC: hepatocellular carcinoma
HE: hepatic encephalopathy
MELD: model for end‐stage liver disease
NPT: neuropsychiatric test
OHE: overt hepatic encephalopathy
OR: odds ratio
SHR: subdistribution hazard ratio

## 1 Introduction

Hepatic encephalopathy (HE) is a common and devastating complication of cirrhosis, which worsens patient morbidity and survival [1]. The pathogenesis of HE is multifactorial and includes systemic inflammation, the gut‐liver axis, and ammonia metabolism, leading to astrocyte swelling and cell death [1, 2]. HE demonstrates a wide spectrum of manifestations from covert HE (CHE) without specific symptoms to overt HE (OHE), which presents as asterixis and coma. CHE is present in approximately 40% of patients with cirrhosis, and OHE develops at an annual incidence rate of 10% among patients with CHE [3, 4]. Even the mildest form of CHE is strongly associated with poor quality of life, frequent falls and accidents, and increased mortality [4, 5]. When CHE progresses to OHE, patients and their caregivers experience impaired social functioning, frequent hospitalizations, and economic burden related to treatment [5]. Therefore, the early detection of CHE and accurate prediction of OHE are essential for optimizing clinical management and reducing the burden on patients with cirrhosis. Neuropsychological tests with test batteries are the reference standard for identifying CHE; however, the time requirement, need for trained practitioners, and associated costs limit their clinical use. Furthermore, even simplified tools such as the Stroop test and animal naming test are not performed in all patients with cirrhosis, reflecting limited medical resources available in real‐world clinical practice [6]. Therefore, developing an accessible and simple method for stratifying the risk of CHE and OHE is essential for establishing effective screening for CHE and careful monitoring of OHE development.

Frailty is a multidimensional syndrome characterized by reduced physiological reserves across the physical, functional, and cognitive domains [7, 8]. In patients with cirrhosis, the prevalence of frailty varies by assessment method, ranging from 17% to 43% [9, 10]. Patients with frailty, particularly those awaiting liver transplantation, have an increased risk of adverse outcomes, including liver decompensation, hospitalization, and mortality [9, 10, 11]. The concept of frailty emphasizes muscle dysfunction, reduced mobility, functional dependence, and cognitive vulnerability, all of which are strongly linked to CHE and OHE [8]. Therefore, the diagnosis of frailty can capture high‐risk populations and serve as an effective method for identifying those at risk for both CHE and OHE. The Clinical Frailty Scale (CFS) is a brief, global frailty assessment tool initially developed for use in the geriatric population [12, 13]. The CFS has also been validated in patients with cirrhosis and is, therefore, recommended by the American Association for the Study of Liver Diseases as a reliable method for identifying frailty [8, 14, 15]. The strengths of the CFS include minimal training requirements, short assessment time, and no necessity for specialized equipment, all of which allow it to be used by non‐specialists in both inpatient and outpatient settings.

Given this background, we hypothesized that frailty assessed using the CFS could help identify patients with cirrhosis at a higher risk of developing HE. This study aimed to evaluate the utility of the CFS in stratifying the risk of CHE and OHE development in patients with cirrhosis.

## 2 Methods

### 2.1 Study Design

This retrospective observational study enrolled patients with cirrhosis who were hospitalized at Gifu University Hospital. The study protocol was reviewed and approved by the Ethics Committee of Gifu University School of Medicine (approval number: 2024–270). This study adhered to the ethical guidelines of the Declaration of Helsinki. Given the retrospective nature of the study, informed consent was obtained using an opt‐out approach.

### 2.2 Participants

This study included hospitalized patients with cirrhosis of any etiology, without a history of OHE or hepatocellular carcinoma (HCC), aged ≥ 18 and < 80 years, and who underwent CHE assessment at Gifu University Hospital between March 2004 and December 2023. Cirrhosis was diagnosed based on the clinical presentation, laboratory data, imaging findings, and liver histology. The exclusion criteria were as follows: history of organ transplantation; active malignancy; neurological or psychiatric disorders; acute systemic diseases, including acute hepatitis, acute‐on‐chronic liver failure, bacterial infection, ruptured varices; life‐threatening comorbidities; large portosystemic shunts; transjugular intrahepatic portosystemic shunt; and opt‐out refusal. All patients received standard medical care in accordance with the guidelines for cirrhosis [16, 17] and were followed up until the last visit, death, or August 21, 2024, whichever occurred first.

### 2.3 Outcomes and Data Collection

The study endpoint was the association between frailty and HE, including the presence of CHE and development of OHE in patients with cirrhosis.

Baseline data were collected from medical records within 1 week of admission and included the following variables: age, sex, body mass index, etiology of cirrhosis, presence of ascites and CHE, and laboratory findings. The etiology of cirrhosis was categorized as viral hepatitis, alcohol‐related liver disease, metabolic dysfunction‐associated steatotic liver disease, or other causes. Ascites was identified using medical imaging. The body mass index was calculated using the estimated dry weight, adjusted by subtracting the percentage of measured weight based on fluid retention: 5% for mild ascites, 10% for moderate ascites, 15% for severe ascites, and an additional 5% for bilateral pedal edema [18].

### 2.4 Diagnosis of CHE and OHE

CHE was diagnosed using a computer‐aided neuropsychiatric test (NPT) conducted within 1 week of admission by trained practitioners, using standardized software (Otsuka Pharmaceutical Co. Ltd., Tokyo, Japan). The NPT comprises four subtests: the number connection tests A and B, digit symbol test, and block design test. CHE was diagnosed when patients exhibited abnormalities in two or more of the four subtests [19, 20]. The NPT is recommended by the Japan Society of Hepatology as the gold‐standard tool for diagnosing CHE and has been validated as a predictor of OHE in Japanese patients with cirrhosis [20, 21]. OHE diagnosis was based on clinical assessment using the West Haven criteria [5].

### 2.5 Diagnosis of Frailty

Frailty was retrospectively assessed by a single hepatologist using the CFS, based on a structured questionnaire of admission‐day medical records, including comorbidities, activities of daily living, and fall risk (Table S1). The CFS comprises nine categories based on the patient's condition (1 = very fit; 2 = fit; 3 = managing well; 4 = very mild frailty; 5 = mild frailty; 6 = moderate frailty; 7 = severe frailty; 8 = very severe frailty; and 9 = terminally ill) [12, 13]. Frailty was defined as a CFS > 4 (CFS 5–9), in line with previously validated criteria [13]. The assessor was fully blinded to CHE and OHE outcome information when performing CFS evaluation.

### 2.6 Statistical Analyses

Baseline characteristics were summarized as numbers with percentages for categorical variables and as medians with interquartile ranges for continuous variables. Comparisons between groups were performed using the chi‐square test for categorical variables and the Mann–Whitney *U* test for continuous variables. Factors associated with the presence of CHE were analyzed using a multivariable logistic regression model, with the results reported as odds ratios (ORs) and 95% confidence intervals (CIs). Considering death as a competing event, the cumulative incidence curves of OHE were estimated using the cumulative incidence function and compared using the Gray's test. In addition, factors associated with OHE occurrence were investigated using the Fine–Gray competing risk regression model, with the results reported as subdistribution hazard ratios (SHRs) with 95% CIs. All statistical tests were two‐sided, with *p‐*values < 0.05 considered statistically significant. Statistical analyses were performed using the R software version 4.4.0 (R Foundation for Statistical Computing, Vienna, Austria).

## 3 Results

### 3.1 Baseline Characteristics of Patients With Cirrhosis

Among the 393 screened patients, 262 met the eligibility criteria and were included in the analysis (Figure S1). The baseline clinical characteristics of the patients are shown in Table 1. The median age was 65 years, 154 (58.8%) patients were female, and the median body mass index was 22.3 kg/m^2^. The most common etiologies of cirrhosis were alcohol‐related liver disease (24.8%), viral hepatitis (22.5%), and metabolic dysfunction‐associated steatotic liver disease (18.7%). The median Child–Pugh, model for end‐stage liver disease (MELD), and albumin‐bilirubin (ALBI) scores were 6, 8, and −2.28, respectively. The median CFS score for the entire cohort was 3. Based on the NPT, CHE was observed in 82 (31.3%) patients.

**TABLE 1:** Baseline characteristics of patients with cirrhosis divided by the presence of frailty.

Characteristic	Overall (*n* = 262)	No frailty (*n* = 237)	Frailty (*n* = 25)	*p* ^a^
Age (years)	65 [55–74]	64 [55–73]	71 [61–75]	< 0.001
Female sex	154 (58.8)	140 (59.1)	14 (56.0)	0.934
Body mass index (kg/m^2^)	22.3 [19.8–25.6]	22.6 [20.1–25.8]	19.6 [18.7–22.5]	0.001
Etiology				0.425
Viral	59 (22.5)	53 (22.4)	6 (24.0)	
ALD	65 (24.8)	57 (24.1)	8 (32.0)	
MASLD	49 (18.7)	45 (19.0)	4 (16.0)	
Others	89 (34.0)	82 (34.6)	7 (28.0)	
Ascites	99 (37.8)	78 (34.0)	21 (84.0)	< 0.001
Varices	110 (42.2)	96 (40.6)	14 (56.0)	0.238
Child–Pugh class				< 0.001
A	145 (55.3)	144 (60.8)	1 (4.0)	
B	68 (26.0)	60 (25.3)	8 (32.0)	
C	49 (18.7)	33 (13.9)	16 (64.0)	
Child–Pugh score	6 [5–9]	6 [5–8]	11 [8–11]	< 0.001
MELD score	8 [7–11]	8 [7–10]	11 [9–16]	< 0.001
ALBI score	−2.28 [−2.67, −1.50]	−2.35 [−2.70, −1.58]	−1.03 [−1.58, −0.57]	< 0.001
Albumin (g/dL)	3.6 [2.9–4.0]	3.7 [3.0–4.1]	2.3 [2.1–2.7]	< 0.001
Bilirubin (mg/dL)	1.1 [0.8–1.6]	1.1 [0.8–1.6]	1.6 [0.9–2.6]	0.026
Creatinine (mg/dL)	0.67 [0.54–0.80]	0.66 [0.54–0.80]	0.79 [0.64–1.05]	0.004
Sodium (mEq/L)	139 [137–140]	139 [137–140]	136 [133–138]	< 0.001
Platelet (10^9^/L)	119 [86–188]	124 [86–188]	102 [73–158]	0.363
International normalized ratio	1.06 [0.99–1.22]	1.05 [0.98–1.17]	1.23 [1.07–1.42]	< 0.001
Ammonia (μg/dL)	56 [39–79]	57 [39–79]	49 [37–80]	0.772
CHE	82 (31.3)	61 (25.7)	21 (84.0)	< 0.001
Clinical frailty scale	3 [3–3]	3 [3–3]	6 [5–6]	< 0.001

*Note:* Values are presented as number (percentage) or median (interquartile range).

Abbreviations: ALBI, albumin‐bilirubin; ALD, alcohol‐associated/related disease; CHE, covert hepatic encephalopathy; MASLD, metabolic dysfunction‐associated steatotic liver disease; MELD, model for end‐stage liver disease.

^a^ Groups were compared using the chi‐square test or Mann–Whitney *U* test.

### 3.2 Comparisons Between Patients With and Without Frailty

**FIGURE 1:**
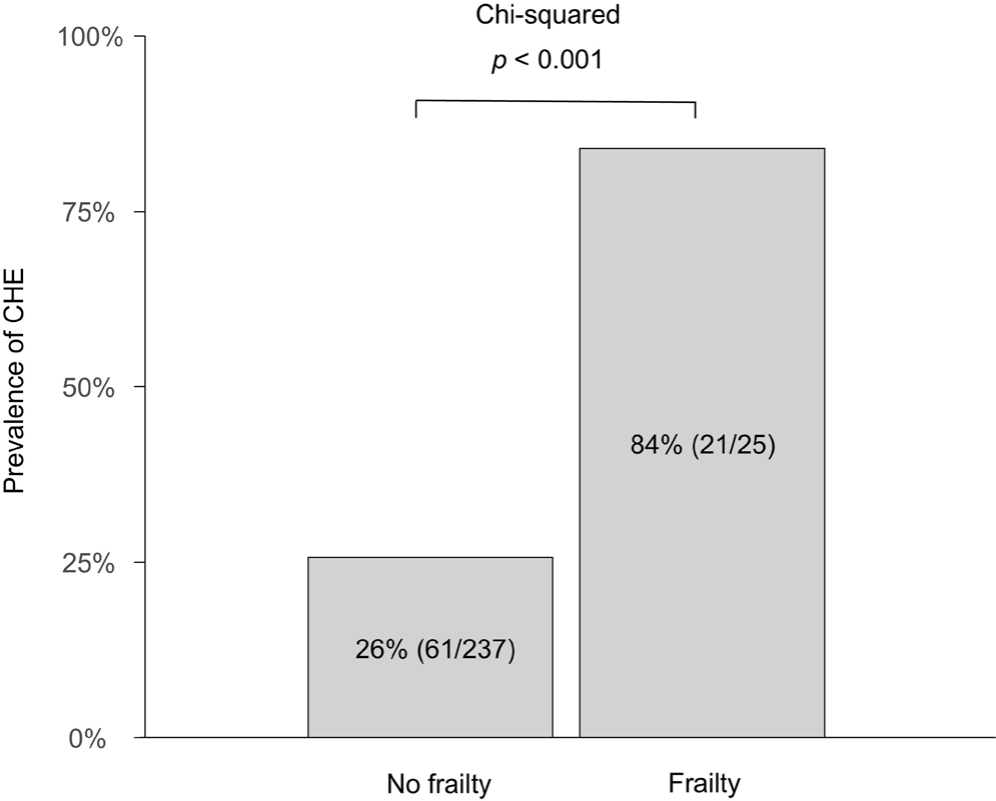
Prevalence of covert hepatic encephalopathy in patients with cirrhosis according to the presence of frailty.

Frailty was identified in 25 (9.5%) patients with a median CFS score of 6 points. Comparisons between patients with and without frailty are presented in Table 1. Compared with patients without frailty, those with frailty had more advanced liver disease, as indicated by higher Child–Pugh, MELD, and ALBI scores, and differences in ascites, albumin, bilirubin, creatinine, sodium, and international normalized ratio. Additionally, patients with frailty had a significantly higher prevalence of CHE than those without frailty (84.0 vs. 25.7%; *p* < 0.001; Figure 1).

### 3.3 Impact of Frailty on the Presence of CHE in Patients With Cirrhosis

Independent factors associated with the presence of CHE in patients with cirrhosis are shown in Table 2. After adjustment, multivariable model 1 identified that CFS (OR, 2.13; 95% CI, 1.41–3.37; *p* < 0.001) and Child–Pugh score (OR, 1.37; 95% CI, 1.19–1.58; *p* < 0.001) were independently associated with CHE. Similar associations were observed in models 2 and 3, which included the MELD and ALBI scores instead of the Child–Pugh score, respectively.

**TABLE 2:** Impact of frailty on CHE in patients with cirrhosis.

Characteristic	Model 1		Model 2		Model 3	
	OR (95% CI)	*p* ^a^	OR (95% CI)	*p* ^a^	OR (95% CI)	*p* ^a^
Age (years)	1.01 (0.99–1.04)	0.366	1.02 (0.99–1.05)	0.247	1.01 (0.98–1.03)	0.609
Male sex	0.95 (0.50–1.76)	0.860	1.01 (0.53–1.93)	0.967	0.99 (0.53–1.84)	0.981
Body mass index (kg/m^2^)	1.00 (0.94–1.07)	0.955	1.03 (0.96–1.09)	0.449	0.99 (0.93–1.06)	0.862
Ammonia (μg/dL)	1.00 (1.00–1.01)	0.114	1.00 (1.00–1.01)	0.439	1.01 (1.00–1.01)	0.160
Albumin (g/dL)			1.07 (0.63–1.83)	0.789		
Child–Pugh score	1.37 (1.19–1.58)	< 0.001				
MELD score			1.12 (1.01–1.26)	0.036		
ALBI score					1.27 (0.90–1.79)	0.173
Clinical Frailty Scale	2.13 (1.41–3.37)	< 0.001	2.69 (1.78–4.30)	< 0.001	2.67 (1.80–4.18)	< 0.001

Abbreviations: ALBI, albumin‐bilirubin; CHE, covert hepatic encephalopathy; CI, confidence interval; MELD, model for end‐stage liver disease; OR, odds ratio.

^a^ Multivariable analyses were performed using the logistic regression model.

### 3.4 Impact of Frailty on the Development of OHE in Patients With Cirrhosis

**FIGURE 2:**
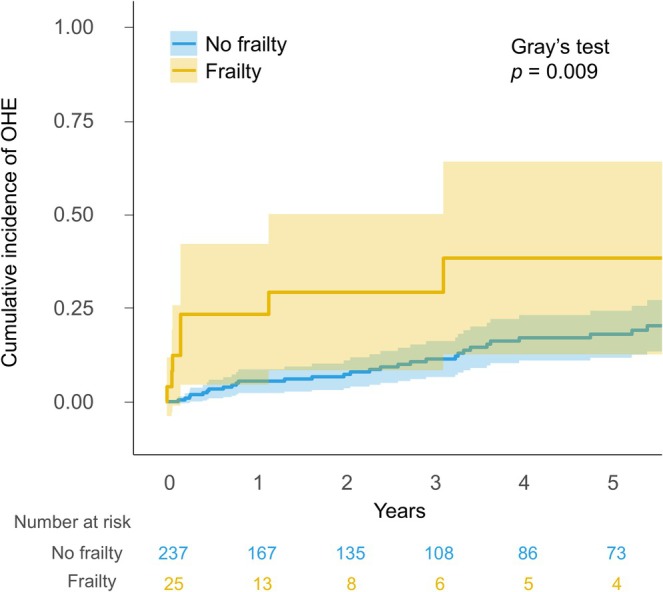
Cumulative incidence of overt hepatic encephalopathy development in patients with cirrhosis according to the presence of frailty.

During the median follow‐up of 2.8 years (interquartile range, 0.8–5.5 years), 40 patients (15.3%) developed OHE, and 20 (7.6%) died. The cumulative incidences of OHE at 1, 3, and 5 years were 7%, 13%, and 20%, respectively in the overall cohort. Patients with frailty had a significantly higher incidence of OHE than those without frailty (median incidence rates at 1, 3, and 5 years; 25%, 33%, and 36% vs. 5%, 11%, and 18%, respectively; *p* = 0.009; Figure 2). Multivariable competing risk regression model 1 showed that the CFS score was an independent predictor of OHE development (SHR 1.38; 95% CI 1.02–1.87; *p* = 0.037; Table 3). Similar findings were observed in models 2 and 3. Additional analysis using the MELD and the serum sodium concentration score showed similar results (Table S2).

**TABLE 3:** Impact of frailty on OHE development in patients with cirrhosis.

Characteristic	Model 1		Model 2		Model 3	
	SHR (95% CI)	*p* ^a^	SHR (95% CI)	*p* ^a^	SHR (95% CI)	*p* ^a^
Age (years)	1.03 (1.00–1.07)	0.069	1.04 (1.00–1.07)	0.056	1.02 (0.99–1.05)	0.300
Male sex	0.81 (0.39–1.67)	0.565	0.66 (0.32–1.37)	0.267	0.78 (0.37–1.67)	0.522
Body mass index (kg/m^2^)	1.00 (0.95–1.06)	0.928	1.00 (0.94–1.07)	0.965	1.00 (0.94–1.07)	0.972
Ammonia (μg/dL)	1.01 (1.01–1.02)	< 0.001	1.01 (1.01–1.02)	< 0.001	1.01 (1.00–1.02)	0.008
Albumin (g/dL)			0.70 (0.39–1.28)	0.246		
Child–Pugh score	1.52 (1.33–1.75)	< 0.001				
MELD score			1.20 (1.08–1.33)	< 0.001		
ALBI score					2.18 (1.57–3.02)	< 0.001
CHE	0.80 (0.36–1.78)	0.584	1.07 (0.49–2.31)	0.868	1.41 (0.67–3.00)	0.367
Clinical Frailty Scale	1.38 (1.02–1.87)	0.037	1.51 (1.12–2.03)	0.006	1.39 (1.00–1.92)	0.047

Abbreviations: ALBI, albumin‐bilirubin; CHE, covert hepatic encephalopathy; CI, confidence interval; MELD, model for end‐stage liver disease; OHE, overt hepatic encephalopathy; SHR, subdistribution hazard ratio.

^a^ Multivariable analyses were performed using the Fine‐Gray model.

## 4 Discussion

The early detection of CHE and accurate prediction of OHE are crucial for the management of cirrhosis. Therefore, simple and easy‐to‐implement tools are urgently required in daily practice. In this study, frailty, assessed using the CFS, detected the presence of CHE. In addition, frailty significantly stratified the risk of developing OHE. Notably, this is the first real‐world study to demonstrate the utility of the CFS in predicting both CHE and OHE in Japanese patients with cirrhosis.

The first key finding is the robust association between frailty and CHE. Physical frailty involves muscle depletion and decreased muscle strength, impairing the ammonia detoxification capacity. The influence of these effects on CHE was previously highlighted in several studies [22, 23]. Beyond muscle loss, frailty reflects a broader construct encompassing not only physical decline but also cognitive and functional vulnerability, which may predispose patients to HE [24]. These factors may explain why frailty, which integrates multiple domains of vulnerability, was a significant predictor of CHE in our cohort. To date, only a few studies have explored the relationship between CFS and CHE. A study in Germany reported that pre‐frailty, as defined by the CFS, was associated with an increased likelihood of CHE diagnosed using the portosystemic encephalopathy syndrome test and animal naming test in patients with and without prior OHE [25]. Studies using physical performance‐based metrics, such as handgrip strength and composite frailty scores, have also linked frailty to CHE [23, 26, 27]. Our findings are consistent with these previous reports and further strengthen the evidence by assessing frailty with the CFS, a validated method for diagnosing frailty; diagnosing CHE using the NPT, a guideline‐recommended diagnostic modality for CHE; and employing the largest cohort of CHE assessments in Japan. Notably, a recent Japanese nationwide survey on HE reported that over 90% of physicians in caring for patients with cirrhosis had not tested even half of their patients, highlighting the critical need to identify the population for whom testing is truly necessary owing to the high risk of CHE [6]. In this context, owing to its simplicity, the CFS is suitable for routine implementation, including in resource‐limited or community settings, and enables the identification of patients at risk of CHE who might otherwise remain unrecognized.

Another key finding was that frailty assessed using the CFS was a robust predictor of OHE development in patients with cirrhosis. Notably, the CFS remained statistically significant even after adjusting for CHE. The independence of CFS and CHE in the development of OHE suggests that frailty may influence the risk of OHE through mechanisms other than those associated with CHE. Patients with frailty may be more vulnerable to common risk factors for OHE development, such as infection, dehydration, and constipation, due to impaired physiological reserve, nutritional deficits, and reduced self‐care capacity [28, 29, 30]. In fact, previous studies have demonstrated that patients with frailty have a higher risk of a composite outcome of liver decompensation than those without frailty [31]. In addition, a prospective cohort study involving 355 patients with cirrhosis demonstrated that a combined score incorporating the CFS and Montreal Cognitive Assessment could stratify the risk of HE‐related hospitalization within 6 months [32]. Furthermore, measurement of handgrip strength, an objective assessment tool for frailty, has also been identified as a predictor of OHE [23]. Our findings, together with the aforementioned reports, reveal the robustness of the association between frailty measurements and OHE development. This is also supported by a German study which found that the CFS predicted OHE occurrence regardless of baseline CHE status [25]. In particular, in the original CFS framework, a score of 4 is defined as “vulnerable” (pre‐frailty) and frailty as CFS > 4, whereas the German study applied a threshold of CFS ≥ 4 to define pre‐frailty [12, 13, 25]. Therefore, different cutoff values for capturing a broader at‐risk population of CHE/OHE require further investigation. Our findings expand the existing knowledge between OHE development and frailty assessed by the CFS, using a large Japanese cohort and robust analysis with adjustment for confounders. Therefore, incorporating frailty assessment into routine clinical evaluation may allow earlier identification of patients at high risk of OHE who require careful observations and timely intervention.

Certain limitations must be considered in this study. First, this cohort consisted of the population of patients assessed for CHE within a specific age range, resulting in a lower prevalence of frailty compared with previous reports [14, 15]. However, a prior study that similarly evaluated CHE reported a frailty prevalence of approximately 11% [25], which is consistent with our findings. This limited prevalence may have reduced statistical power and constrained our ability to fully adjust for residual or unmeasured confounders, including nutrition, inflammation, comorbidities, or medications and interventions during the follow up period. Second, frailty was assessed retrospectively using medical record data rather than through direct evaluation, which may have introduced measurement bias. Third, because this was a single‐center study of hospitalized Japanese patients without acute systemic diseases, the results may not be generalizable to other regions or populations. Finally, there is a strong correlation between frailty and liver functional reserves. Therefore, whether the prognostic value of frailty on OHE development superiors that of liver functional reserves remains unclear.

Despite these limitations, this study also has several strengths. The etiology distribution in this cohort differed from that reported in an earlier Japanese nationwide survey, with a lower proportion of viral hepatitis [33]. This likely reflects both temporal trends, as most patients were enrolled after 2018 when viral hepatitis has declined nationally, and our inclusion criteria excluding patients with prior OHE or HCC. The exclusion of patients with HCC complicating cirrhosis allowed us to analyze a contemporary cohort predominantly composed of alcohol‐related liver disease and metabolic dysfunction–associated steatohepatitis, which represents the recent dynamic change of cirrhosis etiology. Furthermore, the use of a validated global frailty measure, guideline‐recommended ascertainment of CHE, evaluation of both CHE and OHE using competing‐risk methods in a relatively large real‐world cohort, and multivariable adjustment for major confounders collectively strengthen the robustness of our findings. Future prospective studies should examine whether interventions targeting frailty can reduce the risk of CHE and OHE and improve long‐term outcomes in patients with cirrhosis.

In conclusion, frailty, as assessed by the CFS, was a robust predictor of both CHE presence and OHE development. This simple and practical assessment tool may support the early detection of CHE and identification of patients with cirrhosis at high risk of OHE.

## Funding

This study was supported by a Grants‐in‐Aid for Scientific Research from the Japan Society for the Promotion of Science (grant number JP24K18908) and the Japan Agency for Medical Research and Development (grant number JP23fk0210128).

## Ethics Statement

The study protocol was reviewed and approved by the Ethics Review Committee of the Graduate School of Medicine, Gifu University, Japan (approval number: 2024–270).

## Consent

Informed consent was obtained from all study participants using the opt‐out method.

## Conflicts of Interest

The authors declare no conflicts of interest.

## Supporting information


**Figure S1:** Flow diagram of the study.

**Table S1:** Falls and accidents assessment sheet.
**Table S2:** Multivariable model including MELD‐Na score for OHE development in patients with cirrhosis.

## Data Availability

The data and codes used in this study are available from the corresponding author upon request. However, additional approval from the Ethics Review Committee of the Graduate School of Medicine, Gifu University is required to share the data, in accordance with Japanese ethical guidelines.
