# Supplementary figures and images for: Frailty Diagnosed With the Clinical Frailty Scale Stratifies the Risk of Covert and Overt Hepatic Encephalopathy in Patients With Cirrhosis
Source: JGH Open. 2026 Feb 25;10(2):e70369. doi: 10.1002/jgh3.70369 (PMC12935561; doi:10.1002/jgh3.70369)

**Supplementary Fig. 1** Flow diagram of the study

**
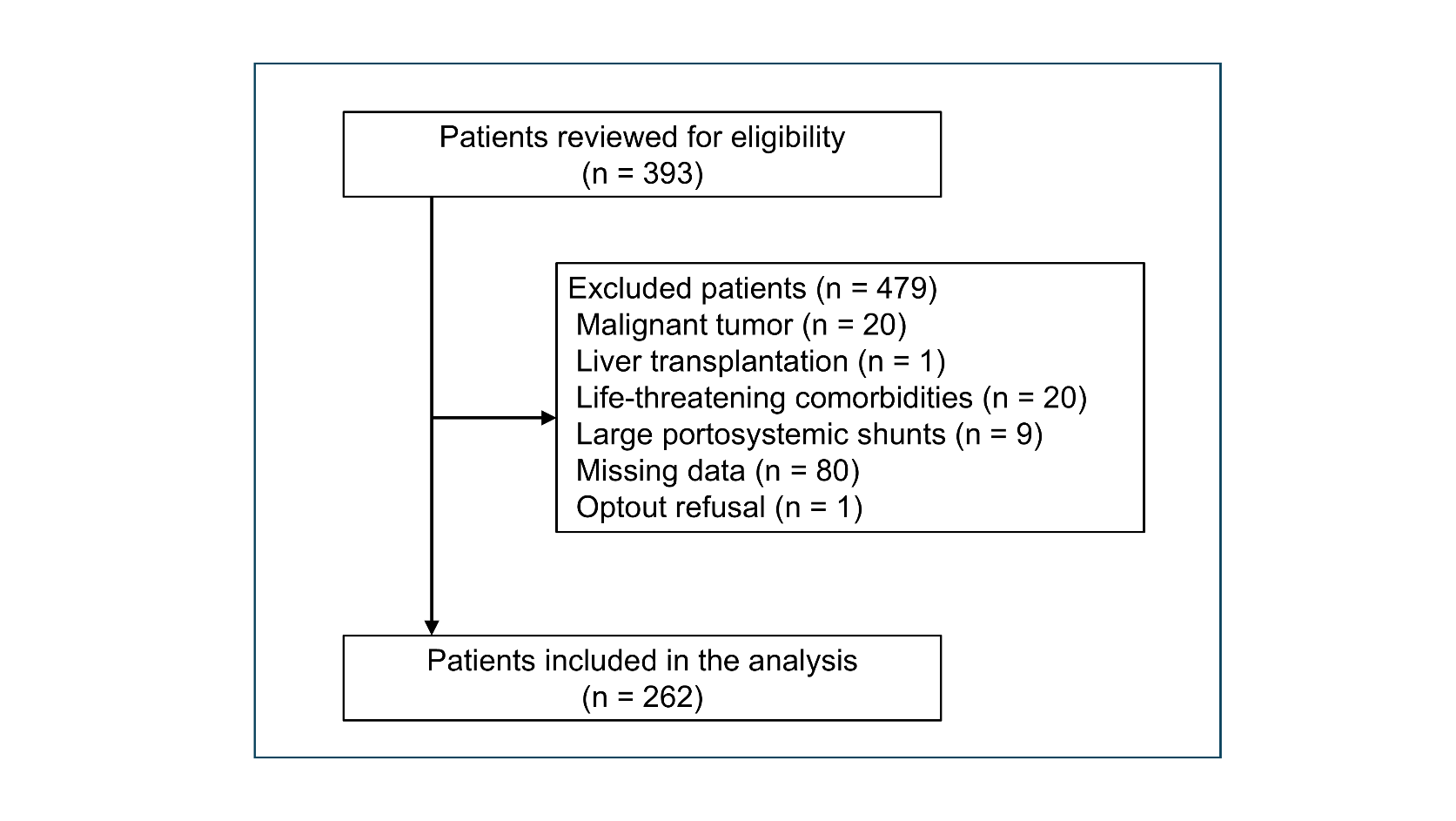
**

Supplement: Supplementary file 1 — Figure S1: Flow diagram of the study. [file JGH3-10-e70369-s002.docx]
