# Supplementary material for: Frailty Diagnosed With the Clinical Frailty Scale Stratifies the Risk of Covert and Overt Hepatic Encephalopathy in Patients With Cirrhosis
Source: JGH Open. 2026 Feb 25;10(2):e70369. doi: 10.1002/jgh3.70369 (PMC12935561; doi:10.1002/jgh3.70369)
Supplement: Supplementary file 2 — Table S1: Falls and accidents assessment sheet. Table S2: Multivariable model including MELD‐Na score for OHE development in patients with cirrhosis. [file JGH3-10-e70369-s001.docx]

Supplementary Table 1. Falls and accidents assessment sheet

| Domain | questions | point |
| --- | --- | --- |
| Age | ☐ Yes (over 70 years old) | 2 |
| History of Falls | ☐ Yes (within the past 1 year) | 2 |
| Cognitive Function | ☐ Exhibits symptoms of cognitive dysfunction  ☐ Exhibits agitated behavior  ☐ Has decreased judgment, comprehension, or memory  ☐ Has disorientation, clouded consciousness, or confusion | 4 |
| Sensation 1 | ☐ Has a neuropsychiatric disorder | 2 |
| Sensation 2 | ☐ Has visual or hearing impairment | 1 |
| Excretion 1 | ☐ Has urinary or fecal incontinence  ☐ Has frequent urination (≥8 times during the day, ≥2 times at night) | 3 |
| Excretion 2 | ☐ Uses a portable toilet  ☐ Uses a wheelchair-accessible toilet  ☐ Requires assistance with excretion | 1 |
| Activity 1 | ☐ Has unsteadiness/gait instability | 3 |
| Activity 2 | ☐ Uses walking aids | 2 |
| Activity 3 | ☐ Requires assistance with mobility  ☐ Is bedridden, but can move their limbs | 1 |
| Motor Dysfunction | ☐ Has paralysis or numbness  ☐ Has bone/joint abnormalities (contracture, deformity) | 1 |
| Patient Characteristic 1 | ☐ Tends to act without pressing the nurse call button  ☐ Cannot recognize or use the nurse call button | 4 |
| Patient Characteristic 2 | ☐ Exhibits independent actions (e.g., restlessness)  ☐ Tries to do everything by themselves | 2 |
| Patient Characteristic 3 | ☐ Has difficulty adjusting to environmental changes (daily life, admission, etc.) | 1 |
| Medical Condition 1 | ☐ Has a fever of 38℃ or higher  ☐ Has anemia  ☐ Prone to lightheadedness (orthostatic hypotension) | 3 |
| Medical Condition 2 | ☐ Within 3 days post-surgery or has drainage tubes inserted | 2 |
| Medical Condition 3 | ☐ In the initial phase of rehabilitation or undergoing training  ☐ Is in a period of rapid recovery or deterioration in symptoms/ADL (Activities of Daily Living). | 1 |
| Medication | ☐ Using one or more of the following medications:  Hypnotics/Sedatives, Tranquilizers, Narcotics, Laxatives, Antihypertensive Diuretics | 4 |
| Total Score | ☐Risk Level I: 0-6 points - Low possibility of falls/accidents  ☐Risk Level II: 7-17 points - Prone to falls/accidents  ☐Risk Level III: 18-39 points - Frequent falls/accidents |  |

Supplementary table 2. Multivariable model including MELD-Na score for OHE development in patients with cirrhosis

| Characteristic | SHR (95% CI) | *p*-value^†^ |
| --- | --- | --- |
| Age (years) | 1.02 (0.99–1.06) | 0.162 |
| Male sex | 0.63 (0.30–1.37) | 0.244 |
| Body mass index (kg/m^2^) | 1.01 (0.95–1.07) | 0.822 |
| Ammonia (µg/dL) | 1.01 (1.01–1.02) | <0.001 |
| Albumin (g/dL) | 0.56 (0.31–1.03) | 0.061 |
| MELD-Na score | 1.09 (1.00–1.18) | 0.040 |
| CHE | 1.17 (0.53–2.59) | 0.706 |
| Clinical Frailty Scale | 1.41 (1.032–1.93) | 0.033 |

†Multivariable analyses were performed using the Fine-Gray model.

Abbreviations: CHE, covert hepatic encephalopathy; CI, confidence interval; SHR, subdistribution hazard ratio; MELD-Na, model for end-stage liver disease and the serum sodium concentration; OHE, overt hepatic encephalopathy
